# Supplementary material for: Demographic patterns of human antibody levels to Simulium damnosum s.l. saliva in onchocerciasis-endemic areas: An indicator of exposure to vector bites
Source: PLoS Negl Trop Dis. 2022 Jan 12;16(1):e0010108. doi: 10.1371/journal.pntd.0010108 (PMC8789114; doi:10.1371/journal.pntd.0010108)
Supplement: S1 File — Fig A–Proportion of tested individuals. Fig B–Age distribution of the population shown per cluster and sex. Table C–Number of people sampled and tested for IgG and IgM per age group and sex. Fig D–IgG antibody distribution according to age shown per cluster. Fig E–IgM antibody distribution according to age shown per cluster. Table F–Summary of exponentiated regression coefficient estimates. Fig F–Correlation between IgG and IgM responses. (DOCX) [file pntd.0010108.s001.docx]

# **S1 File – Supplementary Figures and Tables**

**Demographic patterns of human antibody levels to *Simulium damnosum* s.l. saliva in onchocerciasis-endemic areas: an indicator of exposure to vector bites**

Laura Willen^1,2^ *, Philip Milton^3^, Jonathan I.D. Hamley^3^, Martin Walker^4^, Mike Y. Osei-Atweneboana^5^, Petr Volf^1^, Maria-Gloria Basáñez^3^, Orin Courtenay^6^*

^1^ Department of Parasitology, Faculty of Science, Charles University, Prague, Czech Republic

^2^ Centre for the Evaluation of Vaccinations, Vaccine and Infectious Disease Institute, University of Antwerp, Wilrijk, Belgium

^3^ MRC Centre for Global Infectious Disease Analysis and London Centre for Neglected Tropical Disease Research, Department of Infectious Disease Epidemiology, School of Public Health, Imperial College London, London, UK

^4^ London Centre for Neglected Tropical Disease Research and Department of Pathobiology and Population Sciences, Royal Veterinary College, Hatfield, UK

^5^ Biomedical and Public Health Research Unit, CSIR-Water Research Institute, Accra, Ghana

^6^ Zeeman Institute for Systems Biology & Infectious Disease Epidemiology Research and School of Life Sciences, University of Warwick, Coventry, UK

*[laura.willen@gmail.com](mailto:laura.willen@gmail.com) (LW); [orin.courtenay@warwick.ac.uk](mailto:orin.courtenay@warwick.ac.uk) (OC)

**Fig A – Proportion of tested individuals.** **(a)** Proportion females and males tested per immunoassay. **(b)** Proportion females and males tested per cluster shown for each immunoassay.

**
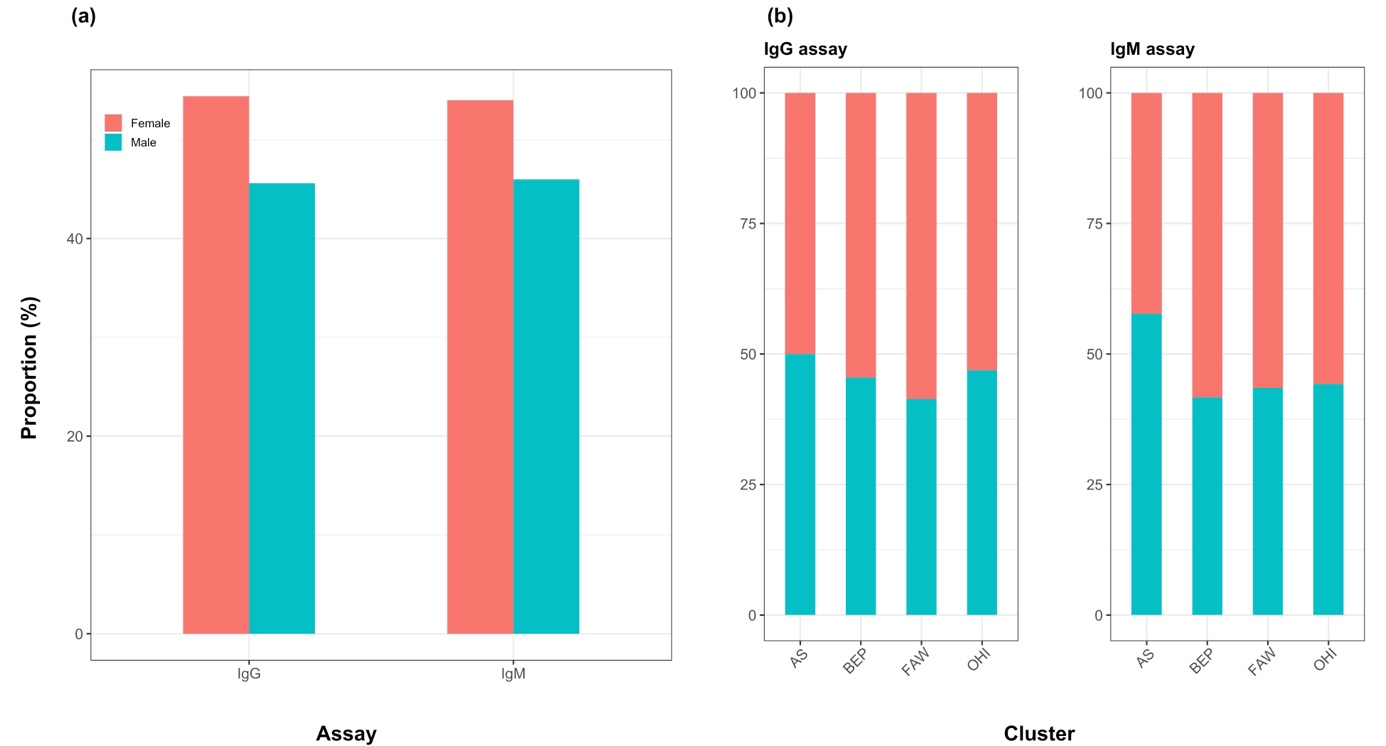
**

AS: Asubende and Senyase; BEP: Beposo; FAW: Fawoman; OHI: Ohiampe.

**Fig B – Age distribution of the population shown per cluster and sex.** Panels **(a)** and **(c)** show boxplots of the age distribution within each cluster, for the participants tested with the IgG and IgM immunoassay, respectively. The solid horizontal line within the boxes is the median; the lower and upper borders are, respectively, the 1st (Q1) and 3rd (Q3) quartiles; the vertical bars indicate the ‘minimum’ and ‘maximum’ values, calculated as Q1 – 1.5 × IQR (interquartile range) and Q1 + 1.5 × IQR, respectively. Panels **(b)** and **(d)** show boxplots of the age distribution per sex, for the participants tested with the IgG and IgM immunoassay, respectively. F: female; M: male. ASU: cluster 1, Asubende/ Senyase; BEP: cluster 2, Beposo; FAW: cluster 3, Fawoman; OHI: cluster 4, Ohiampe.

**
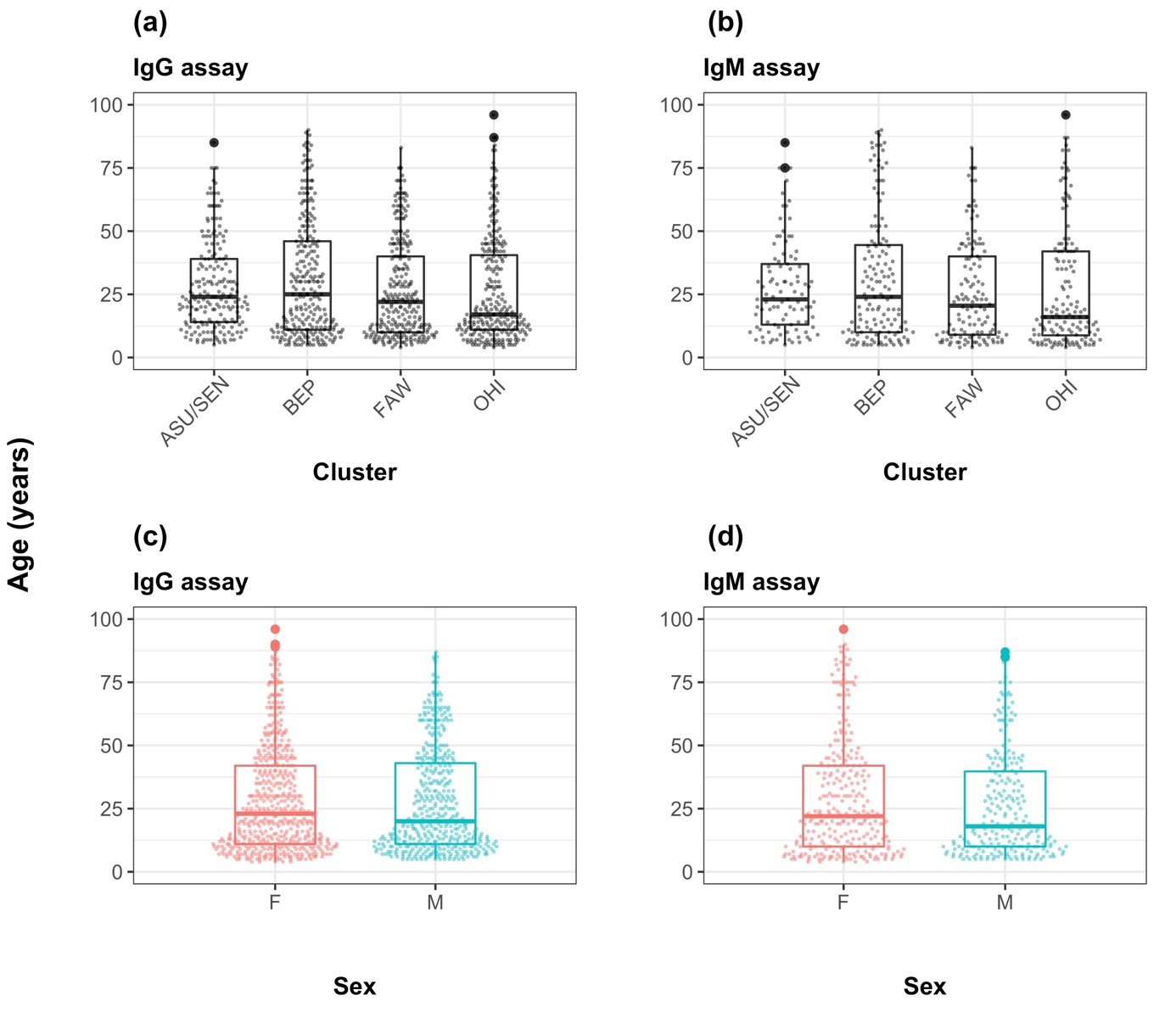
**

**Table C – Number of people sampled and tested for IgG and IgM per age group and sex.**

|  | **Number tested per age group (years)** | | | | | | | |
| --- | --- | --- | --- | --- | --- | --- | --- | --- |
| **Immunoglobulin tested** | **5-10** | **10-20** | **21-30** | **31-40** | **41-50** | **51-60** | **61-70** | **>71** |
| **IgG (n = 958)** | 217 | 252 | 147 | 93 | 101 | 55 | 54 | 39 |
| **IgM (n = 500)** | 140 | 114 | 69 | 50 | 50 | 20 | 20 | 37 |
|  | **Sex ratio (M:F) per age group (years)** | | | | | | | |
| **Immunoglobulin tested** | **5-10** | **10-20** | **21-30** | **31-40** | **41-50** | **51-60** | **61-70** | **>71** |
| **IgG (n = 958)** | 0.87 | 0.92 | 0.63 | 0.75 | 0.91 | 0.96 | 1.35 | 0.44 |
| **IgM (n = 500)** | 0.97 | 0.93 | 0.73 | 0.72 | 0.92 | 0.67 | 1.87 | 0.42 |

n = number, M = Males, F = Females

**Fig D – IgG antibody distribution according to age shown per cluster.** Panel **(a)** shows scatterplots per cluster of the distribution of the IgG responses as a function of age of the participant (shown per sex) with indication of the best-fit lines (solid lines) and their confidence intervals (95 % CI, shown as a coloured area around the line). Panel **(b)** shows boxplots per cluster of the distribution of IgG responses per age group. The solid horizontal line within the boxes is the median; the lower and upper borders are, respectively, the 1st (Q1) and 3rd (Q3) quartiles; the vertical bars indicate the ‘minimum’ and ‘maximum’ values, calculated as Q1 – 1.5 × IQR (interquartile range) and Q1 + 1.5 × IQR, respectively. IgG levels are shown in units/ml. F: female; M: male. ASU: cluster 1, Asubende/ Senyase; BEP: cluster 2, Beposo; FAW: cluster 3, Fawoman; OHI: cluster 4, Ohiampe.


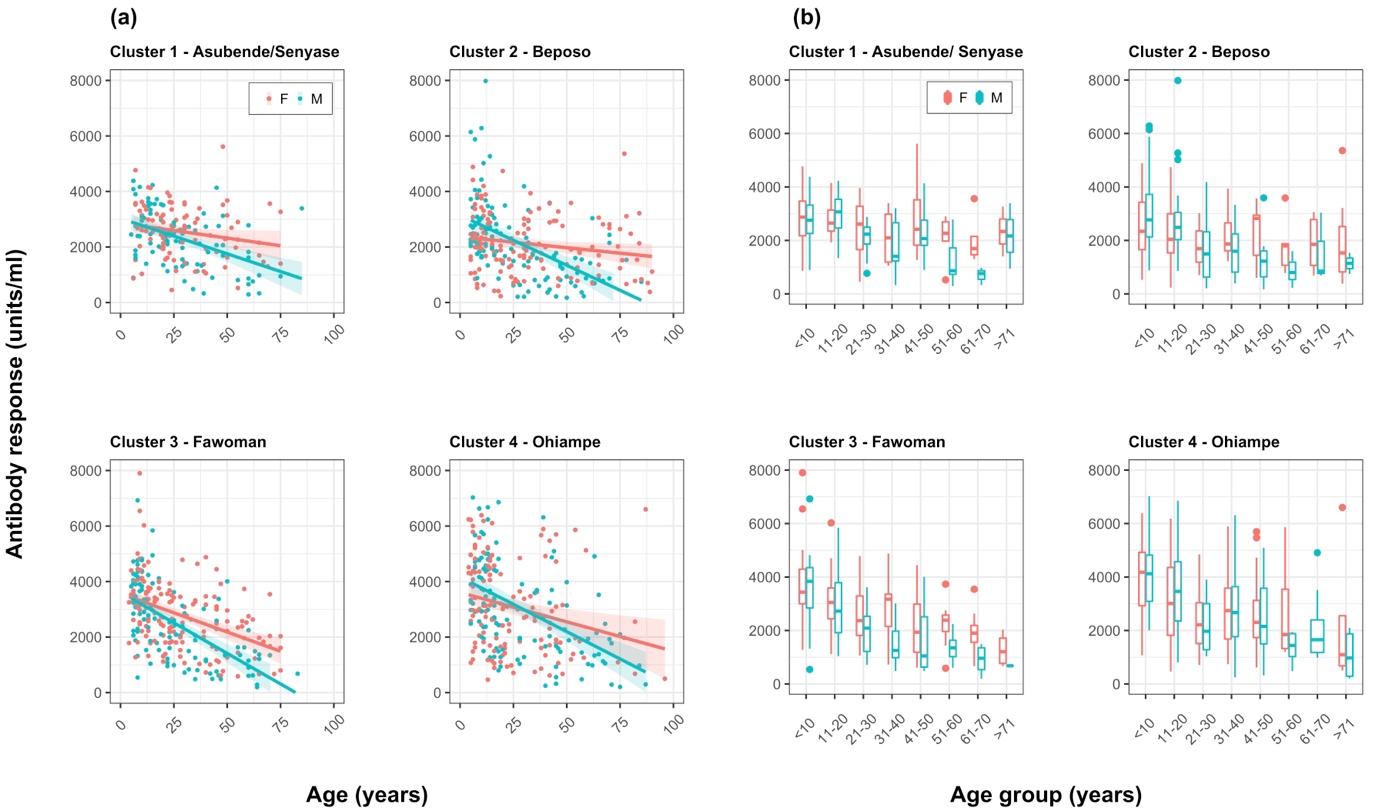


**Fig E – IgM antibody distribution according to age shown per cluster.** Panel **(a)** shows scatterplots per cluster of the distribution of the IgM responses as a function of age of the participant (shown per sex) with indication of the best-fit lines (solid lines) and their confidence intervals (95 % CI, shown as a coloured area around the line). Panel **(b)** shows boxplots per cluster of the distribution of IgM responses per age group. The solid horizontal line within the boxes is the median; the lower and upper borders are, respectively, the 1st (Q1) and 3rd (Q3) quartiles; the vertical bars indicate the ‘minimum’ and ‘maximum’ values, calculated as Q1 – 1.5 × IQR (interquartile range) and Q1 + 1.5 × IQR, respectively. IgM levels are shown in units/ml. F: female; M: male. ASU: cluster 1, Asubende/ Senyase; BEP: cluster 2, Beposo; FAW: cluster 3, Fawoman; OHI: cluster 4, Ohiampe.


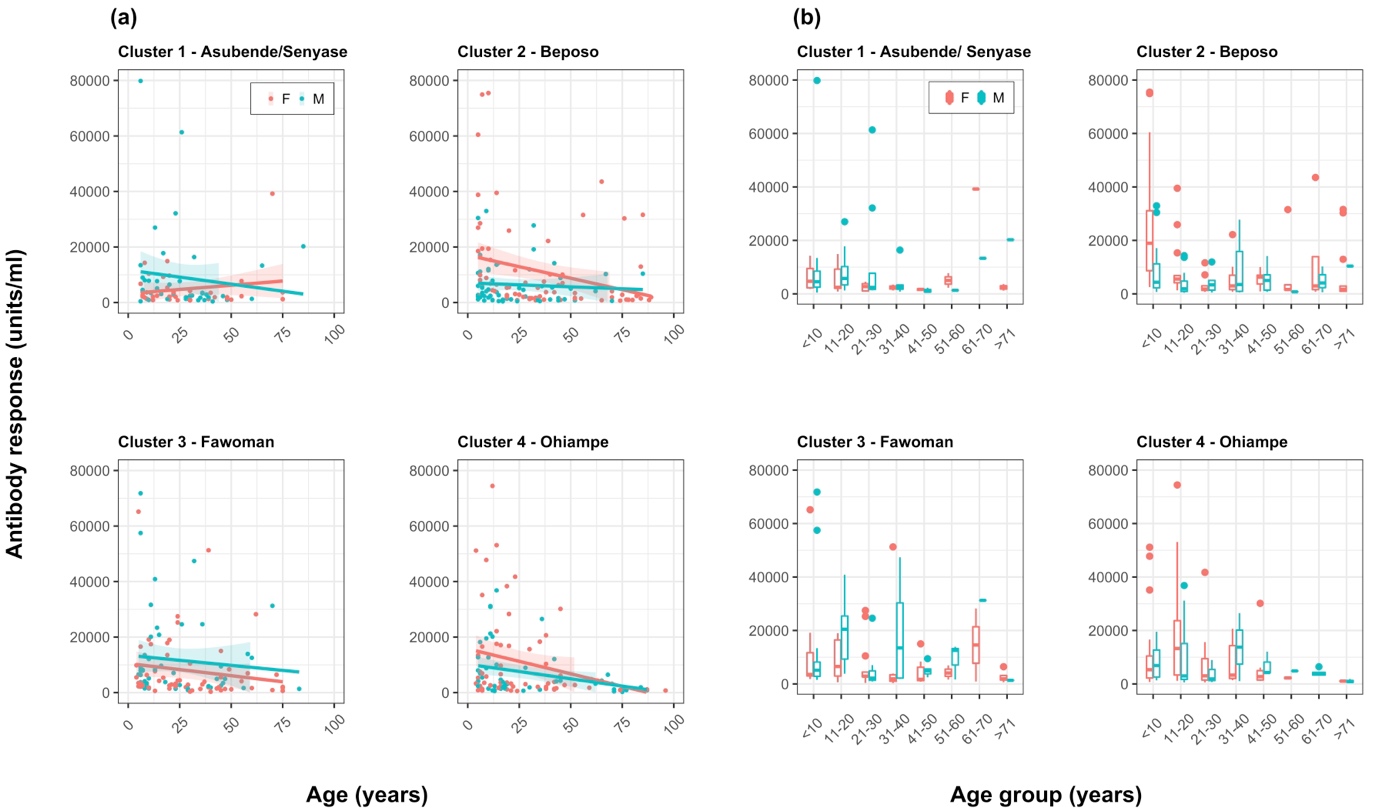


**Table F – Summary of exponentiated regression coefficient estimates.**

|  | **Estimates [95% CI]**  **IgG responses** | **Estimates [95% CI]**  **IgM responses** |
| --- | --- | --- |
| (Intercept) | 2562.291 *** | 7927.734 *** |
|  | [2369.958 – 2770.243] | [5553.306 – 11317.398] |
| BEP | 0.860 ** | 1.191 |
|  | [0.783 – 0.945] | [0.791 – 1.792] |
| FAW | 1.040 | 1.248 |
|  | [0.948 – 1.142] | [0.820 – 1.900] |
| OHI | 1.233 *** | 1.108 |
|  | [1.122 – 1.354] | [0.729 – 1.683] |
| Sex (male) | 0.887 *** | 0.890 |
|  | [0.833 – 0.945] | [0.676 – 1.171] |
| Age | 0.994 *** | 0.988 ** |
|  | [0.992 – 0.996] | [0.980 – 0.996] |
| Age:Sex (male) | 0.990 *** | 1.002 |
|  | [0.987 – 0.993] | [0.990 – 1.015] |

The reference groups were Asubende/ Senyase (ASU/SEN) for village clusters, females for sex and for the age × sex interaction term. Village clusters: BEP: Beposo; FAW: Fawoman, OHI: Ohiampe. 95% confidence interval (CI) shown in square brackets; *** *P*<0.001; ** *P*<0.01; * *P*<0.05.

**Fig F – Correlation between IgG and IgM responses. (a)** shows the overall Spearman correlation between IgG and IgM responses (*r­_s_* = 0.1). **(b)** shows the correlation between both antibody responses broken down by sex (*r­_s_*_males_ = 0.08, *r­_s_*_females_ = 0.12).

**
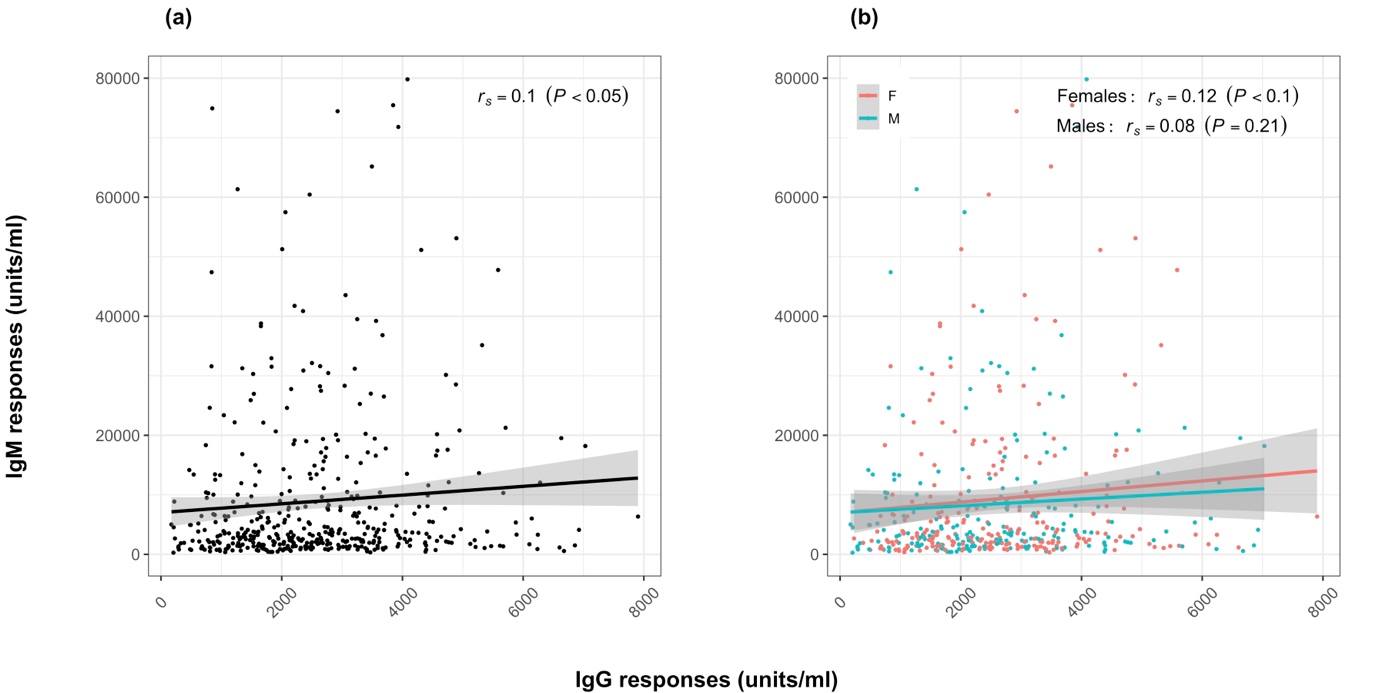
**
