# Supplementary material for: Demographic patterns of human antibody levels to Simulium damnosum s.l. saliva in onchocerciasis-endemic areas: An indicator of exposure to vector bites
Source: PLoS Negl Trop Dis. 2022 Jan 12;16(1):e0010108. doi: 10.1371/journal.pntd.0010108 (PMC8789114; doi:10.1371/journal.pntd.0010108)
Supplement: Attachment — Submitted filename: PNTD-D-21-01444_Review_SW.docx [file pntd.0010108.s002.docx]

**PLOS Neglected Tropical Diseases ( PNTD-D-21-01444)**

**Demographic patterns of human antibody levels to Simulium damnosum s.l. saliva in onchocerciasis-endemic areas: an indicator of exposure to vector bites**

**General comments**

This study is a follow up of the work published by Willen et al. 2021, describing the human immune responses against salivary antigens of *Simulium damnosum* s.l., as new epidemiological marker for exposure to blackflies bite in onchocerciasis endemic areas. The present work used information from previous study and was carried out in four onchocerciasis endemic communities in Ghana. The study objective is to understand sex-or age-related demographic patterns in vector exposure. This study has demonstrated the possibility of sex-and age-specific immunotolerance or desensitization to blackly salivary following the likely cumulative exposure with age. The findings from this pioneer work are likely to contribute to more robust study design that could synchronize microfilarial infection intensity, vector abundance , infection rate and immune responses to blackfly saliva to better understand the transmission risk and intensity and to optimize vector control and surveillance.

**Methods:**

(i)The sample size calculation was based on gender effect and not age effect. Meanwhile the data analysis is more dominated by the age effect, could that influence the findings?

(ii)Logistic regression analysis model could have been more robust to express the association between exposure factors (age, gender, residence, biting rate etc,) and immune responses to Simulium saliva in human. Why the authors did not choose the logistic regression model? I will suggest that the authors redo the data using logistic regression model

**Specific Observations**

L90-98: sentence is too long.

L161: …….kept cool for 2–3 hours……. Describe the cooling system

L114: package of drink powder….. describe better the packaging system

L229-229: “however both IgG and IgM median antibody responses declined with age…. Fig 3D does not seem to reflect that for IgM

L 229-230: A breakdown of the IgG and IgM antibody distribution with age per individual cluster is visualized in Figs D and E in S1 File. There is not figure captioned Fig D and E without a number, do you mean Figs 4 D and E?

L407: replace “cheap” with “ cheaper” and when you say relatively cheaper is compared to what?.

L 407-414: The conclusion needs to be rephrased. say if the initial objective of the study have been reached.
