# Supplementary material for: Demographic patterns of human antibody levels to Simulium damnosum s.l. saliva in onchocerciasis-endemic areas: An indicator of exposure to vector bites
Source: PLoS Negl Trop Dis. 2022 Jan 12;16(1):e0010108. doi: 10.1371/journal.pntd.0010108 (PMC8789114; doi:10.1371/journal.pntd.0010108)
Supplement: Attachment — Submitted filename: Response to Reviewers_15.12.2021.docx [file pntd.0010108.s003.docx]

***PLoS Neglected Tropical Diseases***

Manuscript No. PNTD-D-21-01444

Willen *et al.* Demographic patterns of human antibody levels to *Simulium damnosum s.l.* saliva in onchocerciasis-endemic areas: an indicator of exposure to vector bites

**Editors’ Comments**

All reviewers acknowledged the novelty and quality of this manuscript. Please address the points made by the reviewers, especially expand the discussion on the limitations as mentioned by reviewer #2 and address the comments made by reviewer #3 on sample size calculation in dependence on age and the logistic regression model.

**Reply by Authors to Editors’ comments:** We are very pleased to know that our manuscript has been well received by all three referees and below you will find detailed responses addressing all the points raised. However, since the nature of the data is not binary (we are not measuring seropositivity but antibody levels), a logistic regression model is not appropriate. We have addressed this in **Reply by Authors to 3.2.a** below.

**Response to Reviewers**

**Reviewer #1:**

**1.1)** **General comments:** This is an important study, the first of its kind, and the authors should be congratulated on presenting a clearly written and unfussy manuscript that puts across the key findings in a succinct and accessible manner. My comments are very minor and I have no significant criticisms. However, the raw data have not been made available (or the accession no. is lacking) and no reason is provided for withholding it.

*Reply by Authors to 1.1) We thank the referee for these appreciative comments, and for recognizing the originality of our work. We address the minor comments in detail below. We also agree that the data should be made available and apologize if this was not made clear in our original submission. We have now generated a data repository URL to go live once the article is published, namely,* [*http://wrap.warwick.ac.uk/160693/*](http://wrap.warwick.ac.uk/160693/)

**1.2)** **Methods:** Methods are clearly stated with appropriate statistical analyses supported by power calculations.

*Reply by Authors to 1.2)* *We are pleased to know that the referee thinks our methods are clear and appropriate.*

**1.3)** **Figures and Tables:** Figures and tables are clearly presented and described. However, Table 3 can be deleted as it is redundant with respect to figure 5. In table 2, antibody titres are described as logged but this does not seem to be the case.

*Reply by Authors to 1.3)* *Many thanks for this suggestion.* *Table 3 has been moved to the Supplementary File. Thanks also for noticing the mistake in Table 2; as the referee pointed out the values presented are mean antibody titres, not log-transformed; this has been corrected.*

**1.4)** **Discussion:** The discussion is appropriate and is transparent about the limitations of the study. Could the authors comment on whether the antibody data could be combined with a spatial analysis in future? Notwithstanding the impact of occupation and travel on exposure, it would be expected that biting rates would decline quite rapidly with distance from blackfly breeding sites.

*Reply by Authors to 1.4)* *Thank you for this comment. Yes, indeed it would be very informative if antibody data generated using our immunoassays could be used in spatial analyses to better understand patterns of vector–human contact with increasing distance from breeding sites. At present, mapping exercises such those used for Onchocerciasis Elimination Mapping (OEM) collect information on distribution of breeding sites and vector presence, but our novel tool could complement OEM to identify high-risk locations where exposure to vector bites would provide additional information to Ov16 seroprevalence to guide start-MDA decisions and identify informative age/sex groups for sampling. In addition, most of the information about the relationship between vector density and distance from breeding sites pertains to African savannah settings (such as those explored here), with less data available to characterize such a relationship in forest and forest-savannah mosaic settings. Therefore, if our anti-vector saliva assays could be combined with (seroprevalence) parasite exposure assays for a range of epidemiological settings, it would be possible to obtain very useful information to help elimination efforts. Of course, this would also necessitate the testing and validation of our assays for other species/cytoforms of the* S. damnosum *complex (see also Reply by Authors to 2.4.b).*

*We have included additional text in the Discussion to expand on this as suggested by the reviewer in lines 388-402.*

**1.5) Editorial comments:** The manuscript is very well written, prepared and presented but the reference details should be checked for completeness. Refs. 42 and 47 by "Veriegh FBD" appear to be duplicates and the type of reference is unclear.

*Reply by Authors to 1.5)* *We thank the referee for spotting this. We have now removed duplicates and clarified the reference as a PhD thesis, as follows:*

“Veriegh FBD. Impacts of semi-annual ivermectin treatment and the spatio-temporal distribution and abundance of *Simulium damnosum sensu lato* vectors on the transmission of Onchocerca volvulus. PhD Thesis. Kwame Nkrumah University of Science and Technology (KNUST), Ghana. 2020.”

**Reviewer #2:**

**2.1) General comments:** This is a nice paper with well presented results, but the limitations should be addressed more clearly, and also how the results indicate that more work is needed before the method can be used to help inform transmission models, as so nicely introduced at the start. But the science and writing are good, and with a more detailed discussion of the limitations of the study and the findings the paper will be a good addition to the literature and helps to address the ongoing issue of the HLCs.

*Reply by Authors to 2.1) We thank the referee for these overall positive comments and have endeavoured to address all the points raised as follows.*

**2.2)** **Methods:** Methods are clear and well articulated. A couple of points:

**2.2.a)** Line 145: you say age categories from 5-10, upwards etc. but please amend the recruitment section above to clarify that children 4 and below were not recruited

*Reply by Authors to 2.2.a) This has been added as follows (line 144-145):*

“Children younger than 4 years old were not recruited.”

**2.2.b)** Line 149: I am not sure quite what you mean by: with some variability in percentages tested in the older three age groups. Was this because there were fewer people in these groups?

*Reply by Authors to 2.2.b)* *We have corrected the sentence (lines 150-153). In fact, it was only in the oldest age group where more than the target number (40-60%) was tested for IgM.*

“In the case of IgM, samples were selected by random number generation assigned to the ordered age-stratified full list of samples and aimed to test around 40–60% of the samples tested by IgG per age class, with some variability in percentage tested in the oldest age group.”

**2.2.c)** Line 196: Thank you for the sample size calculations for the IgG, but how did you calculate this for the IgM. The only thing lacking is the explanation of the smaller sample size for the IgM.

*Reply by Authors to 2.2.c)* *The primary aim of the study was to assess the trends in the IgG antibody responses, hence the sample size requirement was calculated and met for IgG. For the addition of detecting trends in IgM responses, we selected a stratified subsample due to the limited availability of antigen. In this case, the statistical power was 90% to detect a difference between sexes with an effect size of D_Cohen_ of 0.3 (as compared to 0.23 for the IgG) and a variance in SOD of 0.16. We have added this to the Methods (lines 206-208):*

“The equivalent statistical power to detect a difference in IgM responses between sexes with an effect size D_Cohen_ of 0.3 and a variance in SOD of 0.16 was 90%.”

**2.2.d)** Line 211: …..were tested, treating….. please include this comma.

*Reply by Authors to 2.2.d) This has been included (lines 215-216):*

“Cluster, sex and age interaction terms were tested, treating age as a continuous variable.”

**2.2.e)** Supplementary figure B1: why were fewer samples from older males used in the IgM ELISAs?

*Reply by Authors to 2.2.e)* *As stated in 2.2.c), the primary aim of the study was to examine the trends in the IgG antibody responses. For the IgM assay we tested only a subsample stratified by age, not sex. Also, overall fewer old people were recruited than younger people, with women being more common than men.*

**2.3)** **Figures:**

**2.3a)** Please say 5-<10 years for all the graphs with the ages on, rather than <10 years

*Reply by Authors to 2.3.a)* *This has been changed in the figures.*

**2.3b)** Please include IgG and IgM in the antibody responses axes labelling, to make it really clear which figure relates to which antibody. As you do for the predicted IgG responses for example.

*Reply by Authors to 2.3.b)* *This has been added to Figs 2 and 3.*

**2.4) Conclusions:** Yes the conclusions are supported, but the limitations should be discussed in greater detail and how this links with public health relevance, in that more work is needed for comparisons, before antibody responses can be used as a proxy for biting exposure.

**2.4.a) Limitations:**

Whilst you discuss that the antibody declines with age could be due to the two very different transmission potential reasons: namely either reducing exposure with age, or maintained high exposure and a reduced antibody response, you should therefore also talk about the limitations that this has on the usefulness of this method to help inform biting rates in models, unless more data are collected to directly link biting rates with saliva antibody responses. You allude to it in the conclusion, but not in the main discussion, so please amend this. You could also talk about the usefulness in children still to assess biting rates in saliva naïve individuals, such as the OV16 type monitoring but for transmission potential should MDA stop.

*Reply by Authors to 2.4.a)* *We agree with the reviewer that more data need to be collected to ascertain the validity of our immunoassays to reliably measure exposure to vector bites. Regarding empirical approaches, there are no experimental or observational data yet to precisely quantify the relationship between anti-salivary antibody levels and the number of vector bites. As colonizing blackflies in the laboratory is notoriously difficult, collecting field data to understand the kinetics of the antibody responses over a specified time frame would be helpful, particularly in settings with strong seasonality, in which vector biting ceases or greatly decreases for several months during the year.*

*Regarding theoretical approaches, and as mentioned in the Discussion, the exposure profiles predicted by age- and sex-structured onchocerciasis transmission models are broadly consistent with the proposed desensitization hypothesis. If this proves to be the case, we expect vector saliva-naïve children still to respond well to salivary antigens as an indication of continued exposure to vector bites pre- or post-MDA campaigns in areas with no vector control or without major ecological changes affecting vector density. In fact, our assay could be used to understand potential secular trends in vector biting rates due to anthropogenic change. Ideally, multiplex assays could be developed that test simultaneously for both exposure to vector bites and exposure to parasite antigens.*

*We have added text to the Discussion to acknowledge these limitations and the need for further data in lines 406-422.*

*We have also revised the Conclusions to better highlight the links between our results and their potential public health relevance as suggested (lines 424-434).*

*Also, we have added text to the first paragraph of the Discussion to further emphasize the importance of obtaining empirical, model-independent data with which to scrutinize exposure assumptions made by current transmission models which are very influential for a number of policy-relevant modelling projections (lines 318-329).*

**2.4.b)** Another further limitation could be that the blackflies used to collect saliva were only from one location. Could this have affected the ELISA antibody assays at all? Is there any evidence that the species might be location specific for reactions at all? As you collected them from the highest transmission site and had the lower responses there maybe this might be another factor in this?

*Reply by Authors to 2.4.b)* *Since the blackflies in the area are all from the same species (*Simulium damnosum*) complex, and all are savannah members of such complex, we do not expect immune responses to differ between different local populations. However, we agree that it is important to compare antibody responses against different species/cytoforms of this complex, as they are the main onchocerciasis vectors throughout Africa, and ascertain if these assays are complex-specific, as we mention in the Discussion (lines 405-406). From our experience with sand flies we know that there are strong cross-reactions between phylogenetically closely-related species.*

**2.5) Editorial comments:** A few very minor changes other than in the methods, results and conclusions described above:

**Abstract/summary**

Line 48: identifying

Line 48: comma after Traditionally,

Line 54: and, rather than or

**Introduction**

First sentence needs splitting into two.

Line 65: please put ‘stop-MDA surveys’ in inverted commas, as not everyone is as used to the term as us.

Line 75: ‘inefficient to’ replace with ‘unable to’

Line 77: please change to ‘…exposure to blackfly vector bites…’

Line 367: greater rate of decline: please be specific in what is declining here?

*Reply by Authors to 2.5) Many thanks for pointing these out. All the suggested changes have been effected.*

**Reviewer #3:**

**3.1) General comments:** This study is a follow up of the work published by Willen *et al*. 2021, describing the human immune responses against salivary antigens of *Simulium damnosum* s.l., as new epidemiological marker for exposure to blackflies bite in onchocerciasis endemic areas. The present work used information from previous study and was carried out in four onchocerciasis endemic communities in Ghana. The study objective is to understand sex-or age-related demographic patterns in vector exposure. This study has demonstrated the possibility of sex- and age-specific immunotolerance or desensitization to blackly salivary following the likely cumulative exposure with age. The findings from this pioneer work are likely to contribute to more robust study design that could synchronize microfilarial infection intensity, vector abundance, infection rate and immune responses to blackfly saliva to better understand the transmission risk and intensity and to optimize vector control and surveillance.

*Reply by Authors to 3.1)* *We thank the reviewer for following our work and for this positive overall assessment of our aims. Indeed, our ultimate objective is to be able to include these assays in monitoring, evaluation, and surveillance activities.*

**3.2) Methods:**

**3.2.a)** The sample size calculation was based on gender effect and not age effect. Meanwhile the data analysis is more dominated by the age effect, could that influence the findings?

*Reply by Authors to 3.2.a) We report the sample size / power calculations for IgG and now IgM to detect differences between sexes. We do not claim to test for differences between specific age groups, hence we do not provide sample size / power calculations for this. The age effect was analyzed using regression analyses to detect trends in Ig responses with increasing age.*

**3.2.b)** Logistic regression analysis model could have been more robust to express the association between exposure factors (age, gender, residence, biting rate etc.) and immune responses to *Simulium* saliva in human. Why the authors did not choose the logistic regression model? I will suggest that the authors redo the data using logistic regression model

*Reply by Authors to 3.2.b)* *The outcome data are not binary; hence logistic regression is not appropriate. The antibody responses are expressed quantitatively in units/ml. To find the appropriate distribution, we fitted several distributions to the units/ml data and assessed this by log-likelihood goodness-of-fit statistics. The best fit was the gamma distribution.*

**3.3)** **Figures / Results:**

**3.3.a)** L229-229: however both IgG and IgM median antibody responses declined with age…. Fig 3D does not seem to reflect that for IgM

*Reply by Authors to 3.3.a)* *This was tested by fitting a univariate GLM (gamma distribution, with a log-link) to the antibody data with age as a continuous variable; this has now been clarified in the Methods (lines 212-215) and formal test (P-values) added to the Results (line 233):*

“Changes in immune responses with age and/or sex were tested by fitting generalized linear models (GLM) to the IgG and IgM anti-blackfly saliva antibody values, where a gamma distribution and log-link function gave the best fit by log-likelihood goodness-of-fit statistics.”

“Equivalent differences were not observed in IgM responses (Fig 3); however, both IgG and IgM median antibody responses declined with age (*P*<0.001) (Fig 2D and 3D).”

**3.3.b)** L229-230: A breakdown of the IgG and IgM antibody distribution with age per individual cluster is visualized in Figs D and E in S1 File. There is not figure captioned Fig D and E without a number, do you mean Figs 4 D and E?

*Reply by Authors to 3.3.b)* *Figs D and E are in the Supplementary (S1) File, not in the main manuscript. The figures in the Supplementary File are not identified by numbers but by letters according to journal style.*

**3.3.c)** The Results section may need improvement based on the previous comments.

*Reply by Authors to 3.3.c) We have addressed all the queries above.*

**3.4) Conclusions:** The conclusion will need to be improved based on the answers to the previous comments.

L407-414: The conclusion needs to be rephrased. Say if the initial objective of the study have been reached.

*Reply by Authors to 3.4) This has been rephrased (lines 426-430) as follows:*

“By novel application to four onchocerciasis-endemic communities, this study successfully evaluated age- and sex-related demographic patterns in blackfly bite exposure. The analyses uncovered the possibility of age- and sex-specific immunotolerance or desensitization to blackfly saliva, likely resulting from cumulative blackfly exposure with age.”

**3.5)** **Editorial comments:**

**3.5.a)** L90-98: sentence is too long.

*Reply by Authors to 3.5.a) The sentence has been slightly reworded and split into two sentences (lines 91-95):*

“This area was under vector control during the Onchocerciasis Control Programme in West Africa (OCP, 1974–2002) [24,25], and in which the first ivermectin community trials were conducted in the late 1980’s [26]. However, transmission persists despite many years of annual MDA, even after adopting biannual MDA in 2010 [27,28].”

**3.5.b)** L161: …….kept cool for 2–3 hours……. Describe the cooling system

*Reply by Authors to 3.5.b) This has been specified (lines 164-165) as follows:*

“and kept cool for 2–3 hours in an insulated cool box until centrifugation at 2500 rpm for 15 min to separate the plasma.”

**3.5.c)** L114: package of drink powder….. describe better the packaging system

*Reply by Authors to 3.5.c) This has been changed to (line 115):*

“a commercial sachet of malt drink powder (Milo, Nestlé®)”.

**3.5.d)** L407: replace “cheap” with “ cheaper” and when you say relatively cheaper is compared to what?

*Reply by Authors to 3.5.d) The word “cheap” has been deleted as we did not perform a cost-effectiveness analysis; the sentence has been changed to (lines 424-426):*

“Serological anti-saliva assays are a useful tool to complement information collected by HLCs by measuring human–vector contact and revealing heterogeneities in exposure at the individual and community level that cannot be unravelled by HLCs alone.”
